# Spatial Patterns of Urban Wastewater Discharge and Treatment Plants Efficiency in China

**DOI:** 10.3390/ijerph15091892

**Published:** 2018-08-31

**Authors:** Min An, Weijun He, Dagmawi Mulugeta Degefu, Zaiyi Liao, Zhaofang Zhang, Liang Yuan

**Affiliations:** 1 Business School, Hohai University, Nanjing 211100, China; anmin@hhu.edu.cn (M.A.); zackzhang@hhu.edu.cn (Z.Z.); 2 College of Economics & Management, Three Gorges University, Yichang 443002, China; liangyuan@hhu.edu.cn; 3 Faculty of Engineering and Architectural Science, Ryerson University, Toronto, ON M5B 2K3, Canada; zliao@ryerson.ca

**Keywords:** spatial pattern, urban wastewater treatment plants, treatment efficiency, data envelopment analysis, exploratory spatial data analysis

## Abstract

With the rapid economic development, water pollution has become a major concern in China. Understanding the spatial variation of urban wastewater discharge and measuring the efficiency of wastewater treatment plants are prerequisites for rationally designing schemes and infrastructures to control water pollution. Based on the input and output urban wastewater treatment data of the 31 provinces of mainland China for the period 2011–2015, the spatial variation of urban water pollution and the efficiency of wastewater treatment plants were measured and mapped. The exploratory spatial data analysis (ESDA) model and super-efficiency data envelopment analysis (DEA) combined Malmquist index were used to achieve this goal. The following insight was obtained from the results. (1) The intensity of urban wastewater discharge increased, and the urban wastewater discharge showed a spatial agglomeration trend for the period 2011 to 2015. (2) The average inefficiency of wastewater treatment plants (WWTPs) for the study period was 39.2%. The plants’ efficiencies worsened from the eastern to western parts of the country. (3) The main reasons for the low efficiency were the lack of technological upgrade and scale-up. The technological upgrade rate was −4.8%, while the scale efficiency increases as a result of scaling up was −0.2%. Therefore, to improve the wastewater treatment efficiency of the country, the provinces should work together to increase capital investment and technological advancement.

## 1. Introduction

Urban wastewater discharge is one of the main sources of surface and groundwater pollution in China [1]. It has exceeded industrial pollution and, since 1998, it has become the largest source of environmental pollution in China [2,3]. With economic development and the intensification of urbanization, the discharge of urban wastewater is increasing year by year. In 2015, urban domestic wastewater discharge was 535.2 billion tons, 4.9% higher than 2014, and accounted for 72.8% of the total wastewater discharge in China [4]. The increase in urban wastewater discharge also raises health risks [5,6]. Furthermore, it threatens the sustainability of the country’s water resources and the socio-economic and environmental systems. Hence, urgent action is needed. Information on the spatial variation of the wastewater discharge and the efficiency of wastewater treatment plants across the country is important for designing a system that can be used to abate the socio-economic and environmental consequences of municipal wastewater emission.

As mentioned above, a clear understanding of the spatial variation of urban wastewater discharge is a prerequisite for reducing water pollution [7]. Most past studies paid attention mainly to the simple description of the spatial variation of urban wastewater treatment efficiency without detailed analysis [8]. Others studied the spatial variations of wastewater discharge [3]. However, research that focuses on analyzing the urban wastewater treatment efficiency and its spatial variation in China is lacking.

Urban wastewater treatment plants (WWTPs) cleanse urban wastewater so that the treated wastewater satisfies the quality requirements for recycled water and controls urban water pollution. The government is paying more attention to urban WWTPs and, as a result, the number and scale of the plants have increased rapidly in recent years [9]. In 2015, the operating cost of wastewater treatment plants in the country was as high as 47.74 billion yuan and was increasing every year. However, at the same time, the operating conditions of urban sewage treatment plants in China have been deteriorating because of problems such as outdated equipment [1]. Therefore, quantitative methods to analyze the efficiency of WWTPs and identify the causes of low efficiency are needed.

The efficiency of urban WWTPs is determined by the level of the technology they operate on and the number of input resources [10]. Water treatment efficiency assessment studies have been done in other countries to diagnose causes for low treatment efficiency. The data envelopment analysis (DEA) model, called CCR (a type of DEA model named after Charnes, Cooper, and Rhodes), was used to analyze the factors responsible for efficiency and inefficiency of 338 sewage treatment plants in Spain [10]. The technical efficiencies of different WWTPs were compared without considering the environmental impact of the wastewater treatment using the DEA frontier model [11]. The efficiencies of Spain’s 99 sewage treatment plants were analyzed with the SBM-DEA (slack-based measure-data envelopment analysis, one type of DEA model) model to quantify the environmental benefits of wastewater treatment [12].

An assessment of the efficiencies of the municipal wastewater treatment plants in China is lacking. However, empirical research to determine the water footprint of different economic sectors in China has been done because this is one of the factors that influence the efficiency of wastewater treatment plants [13,14,15]. The municipal water consumption efficiency was calculated by Bian et al. 2014 [16]. An assessment of the efficiencies of the urban WWTPs is yet to be addressed. Even though the urban wastewater pollution governance is a central government-funded project, each province is autonomous and has independent decision-making power to implement policies. Hence, urban WWTP efficiencies will be determined at a province level.

The systematic quantitative research on urban wastewater discharge and treatment efficiency is of great significance for designing highly efficient urban wastewater treatment schemes. However, studies on this topic are limited. To fill the abovementioned research gaps, in this article we have mapped the spatial variation of urban wastewater discharge and the efficiency of wastewater treatment plants. Our research provides useful suggestions that policymakers can use for improving WWTP urban wastewater remediation efficiency. This can help promote the sustainable development of water resources.

## 2. Materials and Methods

We used the exploratory spatial data analysis (ESDA) method to analyze the spatial variation of urban wastewater discharge and treatment efficiency. Using the input and output data of urban WWTPs, the data envelopment analysis (DEA) model and Malmquist index were used to conduct an in-depth study on the efficiency of urban sewage treatment in mainland China from 2011 to 2015.

### 2.1. Selection of Indicators and Data Sources

The important task when calculating the efficiency of urban wastewater treatment plants is to choose the input and output data. It is determined by considering the data availability and the DEA model‘s input requirement [17,18]. It is also based on the existing literature [10,11,12,19,20,21,22]. The urban wastewater treatment plant number, treatment capacity, and annual operating expenses were taken as input variables. Actual treatment capacity wastewater, removal of chemical oxygen demand (COD) and ammonia nitrogen, which are China’s two major pollutants of water, were the output variables. Since the DEA model does not integrate the indicators, the difference in terms of the units among input and output variables does not matter.

The data of this study were obtained from China’s environmental statistics yearbook (2011–2015) [23] and China Statistical Yearbook (2011–2015) [24]. The annual operating fees were deflated using 2011 as the base year; this was done to eliminate the impact of inflation. The input–output data content are shown in Table 1.

### 2.2. Exploratory Spatial Data Analysis (ESDA) Method

ESDA can depict the spatial characteristics of urban wastewater discharge. We used global spatial autocorrelation (which means the potential variation of wastewater discharge through space) and local spatial autocorrelation to measure this spatial correlation. The Moran’s I index is usually used to determine the variables’ spatial agglomeration and correlation features:

(1) $$I=\frac{n}{\sum_{i=1}^{n}\sum_{j=1}^{n}w_{ij}}\times \frac{\sum_{i=1}^{n}\sum_{j=1}^{n}w_{ij}\left(x_{i}-\bar{x}\right)\left(x_{j}-\bar{x}\right)}{\sum_{i=1}^{n}{\left(x_{i}-\bar{x}\right)}^{2}}$$

where I is the Moran’s I index of the country, n is the total number of provinces included in our study, $x_{i}$ and $x_{j}$ are the urban wastewater discharges of the province i and j, $\bar{x}$ is the mean value of the urban wastewater discharge in each province, and $w_{ij}$ is the spatial weight matrix of province i and province j. Generally speaking, I varies from −1 to 1. If I>0, it indicates a positive spatial autocorrelation. If I<0, it means a negative spatial autocorrelation. When its value is close to 0, it means the economic output is randomly distributed. The bigger the absolute value of I, the greater is the spatial autocorrelation. The significance indicators include P(I), Sd and E(I). P(I) represents the significance level, E(I) is defined as the probability of each possible outcome in the test and Sd is the standard deviation.

Local spatial autocorrelation was used for analyzing the subsystems urban wastewater distribution pattern or spatial heterogeneity. It uses GeoDa software (Center for Spatial Data Science Computation Institute, Chicago, IL, USA) to draw the Moran scatter diagram and measure the interaction among provinces close to each other. The figures in the results section show the four categories and depict the temporal change in each province’s urban wastewater treatment efficiency. The low–low (LL) category indicates that the province’s discharge and its neighboring province’s urban wastewater discharge are low. The high–high (HH) category indicates the opposite. The low–high (LH) category means the province’s urban wastewater discharge is low, but the neighboring province’s is high. The high–low (HL) category is the direct opposite of the LH category.

#### 2.2.1. Super-Efficiency Data Envelopment Analysis (DEA) Model

DEA is a method for evaluating and ranking the relative effectiveness of decision-making units with multiple inputs and outputs. The traditional DEA constructs a frontier or benchmark out of the best practice, which is based on the most efficient units in a given sample. Efficiency is associated with the rational use of all the available resources. It describes the optimal use of all the production factors in a production process [10]. The most efficient units which use minimum inputs to produce a maximum output are called production frontier [25,26]. Other units’ efficiencies can be measured by calculating the maximum possible reduction in the use of inputs [10]. However, when there are many units that belong to the production frontier and their efficiencies are all equal to 1, the traditional DEA model does not show the efficiency differences among these units. The super-efficiency DEA model was established to make up for this defect [27].

The super-efficient DEA model can be described as follows. Given the outputs of each province’s urban WWTPs, the aim of the super-efficient DEA model was to ascertain to what extent the input vectors can be minimized. Efficiency means reducing inputs while inefficiency implies more possibilities of minimizing inputs. It requires solving the following optimization problem by means of linear programming:

(2) $$\begin{array}{l} \min\theta_{jo}\\ s.t.\left\{ \begin{array}{ll} \sum_{j=1}^{n}x_{ij}\lambda_{j}\leq \theta_{j0}x_{in} & i=1,2\ldots m\\ \sum_{j=1}^{n}y_{rj}\lambda_{j}\geq y_{rn} & r=1,2\ldots s\\ \lambda_{j}\geq 0 & \\ j=1,2\ldots n & \end{array}\right. \end{array}$$

Among them, n is the number of decision units, m is the number of input variables, s represents a number of output vectors, $x_{ij}$ and $y_{ij}$ are the input and output vectors’ values, and $\lambda_{j}$ is the dual transform coefficient. Solving this forum and the results of the linear programming $\theta_{j0}$ is the decision units’ urban WWTP efficiency. If $\theta_{j0}\geq 1$, the decision units are production frontiers and greater values indicate higher efficiency.

#### 2.2.2. Malmquist Index

When the time factor is added, the BCC (proposed by Banker, Charnes, and Cooper) and CCR (proposed by Charnes, Cooper, and Rhodes) models of DEA are not capable of measuring the efficiency because the most efficient units change with time. The Malmquist index model (established by Malmquist in 1953) was applied to avoid this drawback [28,29].

The Malmquist index measures the urban WWTP efficiency change. It is also called the total factor productivity change (TFPch). It can be divided into technical efficiency change rate (TEC) and technological progress change rate (TECH) [30]. The procedure is shown below:

(3) $$M_{c}^{t,t+1}\left(x_{t+1,}y_{t+1,}x_{t,}y_{t}\right)=\frac{d_{c}^{t+1}\left(x_{t+1,}y_{t+1}\right)}{d_{c}^{t}\left(x_{t,}y_{t}\right)}\times {\left[\frac{d_{c}^{t}\left(x_{t+1,}y_{t+1}\right)}{d_{c}^{t+1}\left(x_{t+1,}y_{t+1}\right)}\frac{d_{c}^{t}\left(x_{t,}y_{t}\right)}{d_{c}^{t+1}\left(x_{t,}y_{t}\right)}\right]}^{\frac{1}{2}}$$

Among them, $\left(x_{t},y_{t}\right)$ and $\left(x_{t+1},y_{t+1}\right)$ express the input and output at the time periods t and t+1. $d_{c}^{t}$ and $d_{c}^{t+1}$ are the distance functions for the time periods t and t+1. These functions measure the distance of $\left(x_{t},y_{t}\right)$ and $\left(x_{t+1},y_{t+1}\right)$ from the production frontiers.

The technical efficiency change rate (TEC) can be further divided into pure technical efficiency change (PTEch) and scale efficiency change (SEch) [31]:

(4) $$\begin{array}{c} M_{v,c}^{t,t+1}\left(x_{t+1,}y_{t+1,}x_{t,}y_{t}\right)=\frac{d_{v}^{t+1}\left(x_{t+1,}y_{t+1}\right)}{d_{v}^{t}\left(x_{t,}y_{t}\right)}\times \frac{d_{c}^{t+1}\left(x_{t+1,}y_{t+1}\right)/d_{v}^{t+1}\left(x_{t+1,}y_{t+1}\right)}{d_{c}^{t}\left(x_{t,}y_{t}\right)/d_{v}^{t}\left(x_{t,}y_{t}\right)}\\ \times {\left[\frac{d_{c}^{t}\left(x_{t+1,}y_{t+1}\right)}{d_{c}^{t+1}\left(x_{t+1,}y_{t+1}\right)}\frac{d_{c}^{t}\left(x_{t,}y_{t}\right)}{d_{c}^{t+1}\left(x_{t,}y_{t}\right)}\right]}^{\frac{1}{2}} \end{array}$$

Therefore, we have TFPch = TEC × TECH = PTEch × SEch ×TECH.

TFPch is the Malmquist index (WWTPs’ total factor production). TECH is the technological progress, stands for technology innovation and the progress degree. Values more than 1 express progress, less than 1 means decline. When the value equals 1, there is no change. TEC is the index showing the relative change in technical efficiency. Values greater than 1 indicate that the technical efficiency is improving while values less than 1 show the opposite.

## 3. Results and Discussion

### 3.1. Spatial Pattern of Provincial Urban Wastewater Discharge

The spatial variation of nonylphenol, nitrogen, and dichlorodiphenyltrichloroethane (DDT) discharges were studied on a basin scale [32,33,34]. Water quality variation through space was also investigated [35,36]. However, water quality might not give insight into the spatial variation of the wastewater discharge because different water bodies have different dilution capacities. Recently, the spatial characteristics of total wastewater [3] and industrial wastewater discharge [8,37] were assessed. However, studies on urban wastewater discharge are limited.

**Figure 1:**
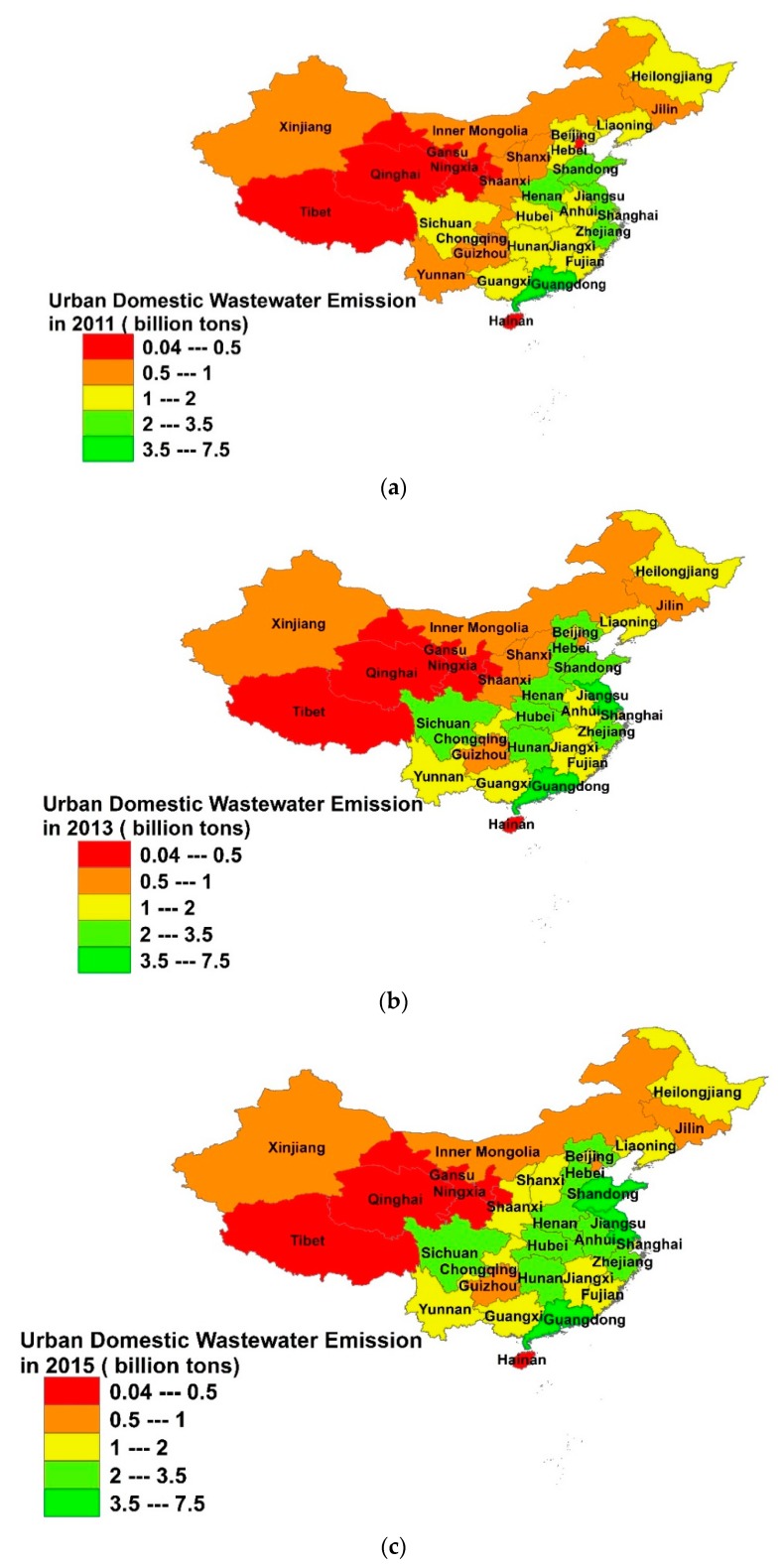
Spatial distribution of urban wastewater discharge in China for (**a**) 2011, (**b**) 2013, and (**c**) 2015. The map only includes China’s 31 mainland provinces. Map generated with ArcGIS 10.6 for desktop.

To fill this research gap, we used the natural breakpoint tool in ArcGIS 10.6 (Esri, Redlands, CA, USA), and we divided the urban wastewater discharge of the 31 provinces for the period 2011–2015 into five levels. Among these levels, the low discharge ranged from 0.04 to 0.5 billion tons, the low and middle discharge ranged from 0.5 to 1 billion tons, the middle urban wastewater discharge ranged from 1 to 2 billion tons, the middle and high from 2 to 3.5 billion tons, and the high discharge ranged from 3.5 to 7.5 billion tons. We chose 2011, 2013, and 2015 as the first, middle, and last year of our study to analyze the spatial variation of the urban wastewater discharge (Figure 1).

**Figure 2:**
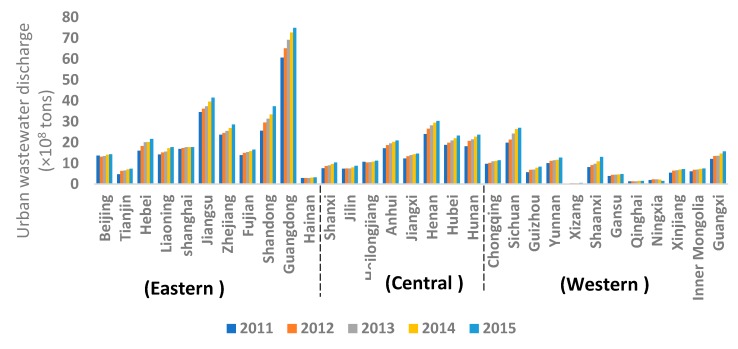
Each province’s urban wastewater discharge for the period 2011 to 2015.

From Figure 1 and Figure 2, it can be seen that the amount of urban wastewater discharge within each province increased from 2011 to 2015. The number of high wastewater discharge provinces also increased. Guangdong was a high-level discharge province throughout the study period, while the urban wastewater discharge from Jiangsu and Shandong provinces increased significantly, making them high discharge provinces gradually. The urban wastewater discharge in Henan, Hebei, Hubei, Hunan, and Anhui also increased considerably.

Relatively high discharge provinces were concentrated in the central and eastern parts of the country. It shows a tendency to spread from the eastern coast to the central and western parts of the country.

The eastern part of China includes most of the economically developed provinces [38]. These provinces’ wastewater discharge was relatively high (Figure 2). Hence, provinces in the eastern part of the country should be pioneers for introducing advanced urban wastewater treatment and management methods, and also should be the leaders in innovating new and improving existing urban WWTP technologies. Policymakers in the provinces located in the central and western parts of China should determine the appropriate rate of urbanization to avoid explosive urban population growth and disorderly urban expansion, which may cause a rapid increase in urban wastewater discharge. In addition, strict environmental standards need to be put in place in these areas [39]. 

The Moran’s I index results (shown in Table 2) describe the spatial relationship among provinces. From Table 2, all the P(I) (Moran’s I index) values passed the 5% significance test. For the period from 2011 to 2015, the value of Moran’s I index remained positive, within the range 0.188–0.176. These values were relatively stable or did not fluctuate that much, indicating that the spatial agglomeration of provincial urban wastewater discharge was relatively stable during the study period.

We used GeoDa software to analyze further the urban wastewater discharge’s spatial agglomeration and identify the provinces that need to implement strict water pollution control because of their intensive urban wastewater discharge. By comparing the Moran scatter diagram for the period from 2011 to 2015, we found that the number of provinces that belong to HH, HL, LH, and LL categories did not change. The results can be seen in Figure 2.

**Figure 3:**
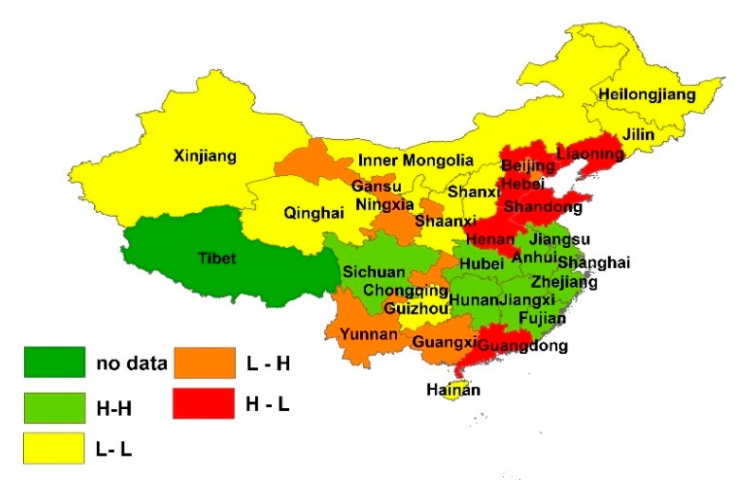
Moran scatter plot for urban wastewater discharge in China. Map generated using ArcGIS 10.6 for desktop.

As shown in Figure 3, the HH category included Jiangsu, Shanghai, Zhejiang, and Fujian provinces, which are gathered in the eastern coastal part of the country, and Anhui, Jiangxi, Hubei, Hunan, and Sichuan provinces, which are located in the central parts of China. The LL category included provinces mainly in the northwestern part of China, such as Xinjiang, Ningxia, Qinghai, and Inner Mongolia. There were also some scattered provinces that belonged to the LL category; among them were Shanxi, Heilongjiang, Jilin, Shaanxi, Guizhou, and Hainan, which were the main ones. These provinces and their neighboring provinces all had low urban wastewater discharge. The HL category included Beijing, Liaoning, Hebei, Shandong, Henan, and Guangdong. The remaining provinces belonged to the LH category and are scattered throughout the country.

All provinces in China should strengthen cooperation. Especially in the regions that contain neighboring provinces that belong to the HH category, inter-provincial linkage mechanisms [40] should be put in place for sharing responsibility and technology transfer among neighboring provinces. This mechanism can serve as a trans-jurisdictional urban wastewater treatment information exchange platform.

### 3.2. The Urban WWTPs Efficiency with Super-Efficiency DEA Model

The above analysis shows the spatial distribution and evolution pattern of urban wastewater discharge, but whether the current urban wastewater treatment plants are enough to control the pollution, needs further study. Even though the WWTP efficiencies were assessed by previous studies [41,42,43,44,45,46], these studies are about specific aspects of WWTPs. Hence, it is not possible to use them as a base for countrywide policy formulation. Since our study was on a provincial scale, it avoids this limitation.

The efficiency of urban wastewater treatment plants in China was calculated using Equation (2). The results are shown in Figure 3, and each province’s urban WWTP efficiency from 2011 to 2015 are presented in the Appendix Table A1.

**Figure 4:**
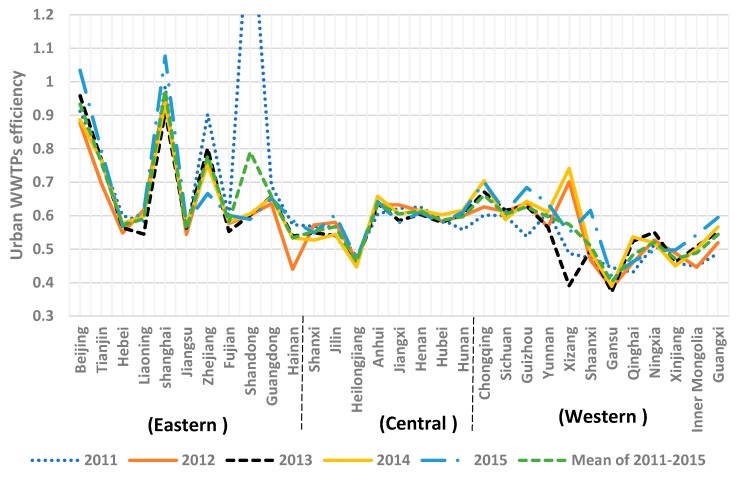
Urban waste water treatment plant efficiency in China from 2011 to 2015.

From Figure 4, the efficiencies of the urban WWTPs of only Shandong in 2011, and Beijing and Shanghai in 2015 were bigger than 1, which means they were the most efficient provinces in terms of wastewater treatment. For Beijing, Tianjin, Shanghai, Zhejiang, Shandong, Guangdong, Anhui, Chongqing, and Guizhou, the average efficiency for the five years was above the overall mean. This is because these regions are economically developed and have advanced urban wastewater treatment technology. Generally, the efficiencies of the urban WWTPs decreased between 2011 and 2013 and then increased between 2013 and 2015. The country’s overall average efficiency for the five years was 0.608 (Appendix Table A1). This indicates that China’s overall urban WWTP efficiency has a great potential to improve since the loss of urban WWTP efficiency was as high as 39.2%. The reason for this low efficiency is the fact that the resources allocated to controlling water pollution were relatively insignificant.

The efficiencies of urban WWTPs of the eastern part of the country were greater than the central part of the country and western part of the country for the study period (Figure 3). The efficiency of the central part of the country was greater than the western region of the country. East China is the more developed region with a high level of urbanization, industrial structure, and high technological advancement. This is the main factor that made WWTP efficiency in the eastern region better.

However, the efficiency of the eastern part of the country in 2015 was 0.705 (Appendix Table A1), lower than its efficiency in 2011, which was 0.791 (Appendix Table A1). This indicates urban WWTP efficiency declined in provinces located in the eastern part of the country, and the technological upgrade did not keep up with the development of socio-economic systems. The efficiencies of urban WWTPs in cities located in the central and western parts of the country were higher in 2015 than in 2011. If this trend were to continue, the superiority of the eastern part of the country in terms of urban WWTP efficiency would disappear.

According to the analysis above, more and more attention should be paid to water pollution remediation, especially by investing capital that can keep up with the economic and population growth. The eastern regions should play a technological spillover effect so that the central and western provinces improve their wastewater remediation efficiency. Provincial governments should, according to their urbanization process, reasonably adjust the scale and financial investment allocated to urban wastewater treatment.

### 3.3. The Results of the Malmquist Index

**Figure 5:**
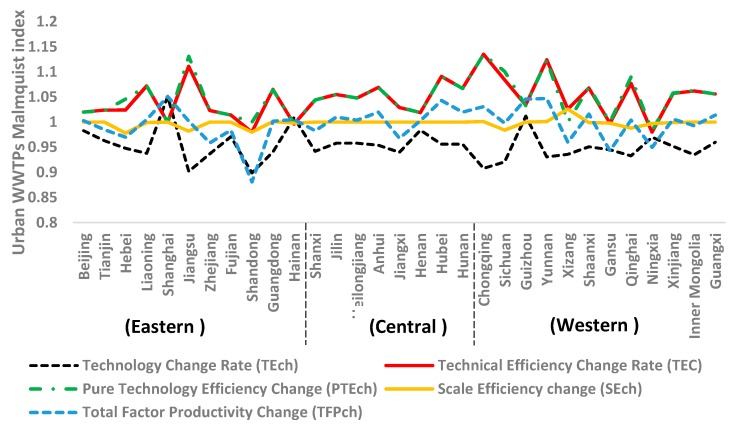
The average change in urban WWTP efficiency for the period from 2011 to 2015.

Based on the urban WWTPs’ input and output data of China’s 31 provinces and using Equations (3) and (4), the average urban WWTP efficiency and efficiency change for the period from 2011 to 2015 was calculated. The results are shown in Figure 5 and the results of each province are shown in Appendix Table A2. The total factor productivity change (TFPch) is divided into technological change rates (TEch) and technical efficiency change rate (TEC). If the value of TFPch is greater than 1, there was technological progress. The technical efficiency changes rate (TEC) >1 indicates that technical efficiency was improved.

According to Appendix Table A2, the average urban WWTP efficiency change (TFPch) for China in the period from 2011 to 2015 was 0.996, which means that urban WWTP efficiency had a negative growth. This was mainly due to the negative growth of the technological change rate (TEch), which was −4.8%, and the scale efficiency change (SEch), which was −0.2%.

As it can be seen from Figure 5, the technological change rate (TEch) in the eastern, central, and western regions was obviously insufficient for urban WWTPs. Only Shanghai, Hainan, and Guizhou’s TEch improved between 2011 and 2015. For the other provinces, the contrary happened. These provinces should focus on improving the efficiency of their WWTPs by increasing capital investment.

The scale efficiency change (SEch) was on a decline in the recent years, especially in the eastern part of the country (Figure 5). As a result, the number of WWTPs in the region has not been increasing at a rate that can keep up with the wastewater discharge. Provinces such as Hebei, Liaoning, Jiangsu, Shandong, Hainan, Sichuan, Shaanxi, Gansu, Qinghai, Ningxia, and Xinjiang also have the same problem.

Urban WWTP efficiency with regards to TEC and PTEch increased in the central and western parts of the country between 2011 and 2015. This was mainly due to technological improvements. In addition, improved management of wastewater treatment plants also contributed to the increase in efficiency. Hainan’s, Shandong’s and Ningxia’s technical efficiency change rate (TEC) declined; hence, these three provinces should focus on improving the efficiency of their municipal wastewater treatment plants.

Generally, efficient urban wastewater remediation protects water bodies and promotes sustainable development. Hence, the following measures could help in building efficient wastewater remediation systems. First, provinces located in the eastern part of the country should form a water pollution control alliance with provinces in the central and western parts of China. This would allow for the joint development of new technologies and cost sharing. Second, the government should increase investment in new wastewater treatment plants and also pay attention to the renewal of old treatment facilities to maintain efficiency. Each province should increase the technical and scale of their urban sewage treatment plants according to their different situations. Third, the government should establish a reasonable urbanization speed and plan the scale of their urban WWTP facilities accordingly. Fourth, the government needs to organize technical exchanges and seminars and strengthen the technical training for operational management personnel to improve the overall level of wastewater remediation. Last, the public should vigorously advocate for water conservation, social supervision, and participation, as well as for the reduction of urban wastewater discharge at the source.

Our proposed framework could be modified and improved by addressing the following research gaps through further study. First, to increase the resolution of the assessment, the model needs to be applied on a smaller spatial scale, such as a county. Second, additional factors that influence urban WWTP efficiency should be taken into account. Third, the time span for the study period should be increased.

## 4. Conclusions

Urban wastewater discharge has become a growing problem that is restricting sustainable development in China. In this research, the authors attempted to capture the spatial pattern of urban wastewater discharge and the efficiency of urban wastewater treatment plants across the 31 provinces of mainland China for the period from 2011 to 2015. The main conclusions that are extracted from the results are the following. (1) Urban wastewater discharge is increasing and has obvious spatial and temporal variations that policymakers should consider when formulating urban WWTP treatment policies. (2) The urban WWTP efficiency in China is low, but the efficiency of urban WWTPs located in the eastern part is relatively better than that of provinces located in the central and western parts of the country. (3) To address the different WWTPs’ efficiencies from province to province, common but differentiated urban WWTP policies should be formulated. Taking these measures will bring equity among the provinces in terms of their urban waste water remediation efficiency.

## Figures and Tables

**Table 1:** Input and output variables of urban wastewater treatment plants in China for the time span 2011 to 2015.

Statistical Description	Input Indicators			Output Indicators		
	Number of Urban Wastewater Treatment Plants	Treatment Capacity of Urban Wastewater Treatment Plant (×10,000 tons/day)	Annual Operating Expenses (×10,000 CNY)	Actual Treatment Capacity of Urban Wastewater (×10,000 tons)	Removal of Chemical Oxygen Demand from Urban Wastewater (ton)	Removal of Ammonia from Urban Wastewater (ton)
Mean	173.59	531.28	120,554.43	148,497.81	360,704.25	32,773.86
Maximum	797	2329	524,447.11	709,942	1,470,883.1	146,396
Minimum	2	1	162	236	532.6	40.1
Median	134	383	87,567.2	102,321	239,205.8	22,648.4
Standard Deviation	149.94	455.72	117,992.56	136,909.03	336,531.03	29,502.38
Number of Observations	155	155	155	155	155	155

**Table 2:** Global Moran’s I test of urban wastewater discharge in China for the period from 2011 to 2015.

Year	Moran’s I	E(I)	Sd.	P(I)
2011	0.188	−0.034	0.106	0.018
2012	0.183	−0.034	0.106	0.020
2013	0.179	−0.034	0.106	0.022
2014	0.18	−0.034	0.106	0.022
2015	0.176	−0.034	0.106	0.025

## Appendix A

**Table A1:** Changes in urban wastewater treatment plant (WWTP) efficiencies in China from 2011 to 2015.

	Time	2011	2012	2013	2014	2015	Mean
Province							
Beijing		0.912	0.877	0.958	0.886	1.034	0.933
Tianjin		0.772	0.697	0.767	0.765	0.796	0.759
Hebei		0.6	0.548	0.564	0.577	0.568	0.571
Liaoning		0.582	0.617	0.545	0.598	0.613	0.591
shanghai		0.985	0.96	0.909	0.937	1.077	0.974
Jiangsu		0.545	0.544	0.563	0.57	0.578	0.56
Zhejiang		0.903	0.767	0.801	0.752	0.666	0.778
Fujian		0.593	0.577	0.553	0.581	0.601	0.581
Shandong		1.552	0.601	0.602	0.605	0.589	0.79
Guangdong		0.689	0.636	0.658	0.655	0.652	0.658
Hainan		0.573	0.44	0.539	0.533	0.585	0.534
Eastern average		0.791	0.660	0.678	0.678	0.705	0.703
Shanxi		0.568	0.572	0.549	0.527	0.549	0.553
Jilin		0.566	0.58	0.541	0.544	0.603	0.567
Heilongjiang		0.477	0.467	0.455	0.447	0.469	0.463
Anhui		0.605	0.633	0.636	0.658	0.642	0.635
Jiangxi		0.622	0.632	0.585	0.605	0.579	0.605
Henan		0.626	0.614	0.604	0.616	0.61	0.614
Hubei		0.589	0.583	0.58	0.602	0.578	0.586
Hunan		0.558	0.599	0.603	0.616	0.611	0.597
Median average		0.577	0.585	0.569	0.577	0.580	0.578
Chongqing		0.602	0.626	0.671	0.704	0.7	0.661
Sichuan		0.6	0.612	0.616	0.588	0.611	0.605
Guizhou		0.538	0.638	0.629	0.643	0.684	0.626
Yunnan		0.611	0.565	0.565	0.61	0.636	0.597
Xizang		0.487	0.701	0.391	0.741	0.549	0.574
Shaanxi		0.474	0.468	0.497	0.493	0.616	0.51
Gansu		0.445	0.384	0.372	0.389	0.418	0.402
Qinghai		0.431	0.46	0.524	0.536	0.463	0.483
Ningxia		0.513	0.527	0.551	0.519	0.496	0.521
Xinjiang		0.458	0.488	0.461	0.45	0.498	0.471
Inner Mongolia		0.449	0.446	0.508	0.501	0.543	0.489
Guangxi		0.488	0.519	0.552	0.566	0.595	0.544
Western average		0.508	0.536	0.528	0.562	0.567	0.54
Total average		0.626	0.593	0.592	0.607	0.62	0.608

**Table A2:** The average changes of urban WWTP efficiency change for the period from 2011 to 2015.

Productivity	Technology Change Rate (TEch)	Technical Efficiency Change Rate (TEC)	Pure Technology Efficiency Change (PTEch)	Scale Efficiency Change (SEch)	Total Factor Productivity Change (TFPch)
Beijing	0.983	1.020	1.020	1.000	1.003
Tianjin	0.963	1.024	1.023	1.000	0.985
Hebei	0.948	1.024	1.046	0.978	0.970
Liaoning	0.938	1.072	1.072	0.999	1.005
Shanghai	1.051	1.000	1.000	1.000	1.051
Jiangsu	0.902	1.111	1.131	0.982	1.002
Zhejiang	0.937	1.023	1.023	1.000	0.959
Fujian	0.971	1.014	1.014	1.000	0.984
Shandong	0.899	0.980	1.000	0.98	0.881
Guangdong	0.941	1.065	1.064	1.001	1.002
Hainan	1.009	0.996	0.998	0.998	1.005
Eastern average	0.958	1.030	1.036	0.994	0.986
Shanxi	0.942	1.044	1.044	1.000	0.983
Jilin	0.958	1.055	1.055	1.000	1.010
Heilongjiang	0.958	1.048	1.048	1.000	1.004
Anhui	0.954	1.069	1.069	1.000	1.020
Jiangxi	0.940	1.029	1.030	1.000	0.968
Henan	0.984	1.019	1.019	1.000	1.003
Hubei	0.956	1.091	1.091	1.000	1.043
Hunan	0.956	1.067	1.067	1.000	1.020
Median average	0.956	1.053	1.053	1.000	1.006
Chongqing	0.908	1.135	1.135	1.001	1.031
Sichuan	0.921	1.084	1.101	0.984	0.998
Guizhou	1.012	1.034	1.033	1.000	1.046
Yunnan	0.931	1.124	1.124	1.001	1.047
Xizang	0.936	1.026	1.000	1.026	0.960
Shaanxi	0.951	1.068	1.070	0.999	1.016
Gansu	0.945	0.998	1.000	0.998	0.943
Qinghai	0.933	1.077	1.090	0.988	1.004
Ningxia	0.970	0.980	0.983	0.997	0.950
Xinjiang	0.952	1.057	1.058	0.999	1.006
Inner Mongolia	0.935	1.062	1.063	1.000	0.993
Guangxi	0.960	1.056	1.056	1.000	1.014
Western average	0.946	1.058	1.059	0.999	1.001
Total average	0.952	1.046	1.048	0.998	0.996

Note: According to Equation (3), TFPch = TEC × TEch = PTEch × SEch × TECH. Therefore, in this Table A2, TEC = PTEch × SEch, TFPch = TEch × TEC.
